# A real-time virtual machine for task placement in loosely-coupled computer systems

**DOI:** 10.1016/j.heliyon.2019.e01998

**Published:** 2019-06-27

**Authors:** Mohamed O. Elsedfy, Wael A. Murtada, Ezz F. Abdulqawi, Mahmoud Gad-Allah

**Affiliations:** a Military Technical College, Cairo, Egypt; b National Authority for Remote Sensing & Space Sciences, Cairo, Egypt; c Vice Dean of Modern Academy, Cairo, Egypt

**Keywords:** Computer science, Task placement, Process virtual machine, Language translation, Loosely-coupled, Real-time

## Abstract

Nowadays, virtualization and real-time systems are increasingly relevant. Real-time virtual machines are adequate for closely-coupled computer systems, execute tasks from associated language only and re-target tasks to the new platform at runtime. Complex systems in space, avionics, and military applications usually operate with Loosely-Coupled Computer Systems in a real-time environment for years. In this paper, a new approach is introduced to support task transfer between loosely-coupled computers in a real-time environment to add more features without software upgrading. The approach is based on automatic source code transformation into a platform-independent “Structured Byte-Code” (SBC) and a real-time virtual machine (SBC-RVM). Unlike Ordinary virtual machines which virtualize a specific processor for a specific code only, SBC-RVM transforms source code from any language with a known grammar into SBC without re-targeting the new platform. SBC-RVM executes local or placed tasks and preserving real-time constraints and adequate for Loosely-coupled computer systems.

## 1 Introduction

Complex real-time systems; such as satellites, nuclear power plants, military, and aerospace control systems, are designed for long-term operations and strict timing requirements. These complicated and costly systems shall be in service for years without a significant upgrade. For instance, a significant software update may cause a catastrophic problem like in the case of “X-ray Astronomy Satellite “Hitomi” (ASTRO-H)” anomaly. The communication was lost with Hitomi when an in-orbit software update was being uploaded [1]. This kind of systems are characterized by reliability, predictability, and heritage of operation, whereas they do not rely on the fast progress at integrated circuits speed, novel processors architectures and the number of cores. The software for such systems is designed for a specific platform to achieve the desired parameters such as frequency, priority, worst-case execution time, bounded jitter, energy, and cost.

### 1.1 Problem statement

The particular constraints are real-time systems which operate continuously in a harsh environment for years. This research focuses on loosely coupled computer systems. The considerable system is space-systems such as satellites. In-system programming is a critical operation, whereas operation for long life without updates is inferior. The requirement to add new features without software upgrading is highly needed. Subsystems from different vendors with various platforms and RTOSs should be able to communicate not for exchanging data but also exchanging tasks. Exchanging tasks between subsystems for load balancing and fault tolerant can improve system reliability and hence a commonly spoken language, execution platform, and support framework are required.

### 1.2 Proposed approach

The dilemma among long-term operations and upgrading cost for that kind of systems can be resolved by using a platform-independent real-time virtual machine, which accepts the old and new developed code, supports task placement between nodes on the network and remote command-execution while preserving real-time constraints.

A real-time virtual machine called structured byte code (SBC- RTVM) is introduced. SBC- RTVM is based on three principles that are: source-code/SBC-code automatic generation, SBC platform independence, and real-time task properties conservation. SBC-RTVM can exchange and execute source code written in a different language for different platforms while preserving origin real time constraints. SBC generation, SBC-RTVM architecture, and inter-process communications among heterogeneous loosely-coupled computers are introduced. The proposed solution is best appropriate in satellite OBC with other subsystems.

ANSI C, C++, Python, and Java are the most common programming languages used in such real-time systems. This research will focus on ANSI C language which considered as one of the most used languages in such complex systems.

The paper is structured as follows. Section 2 discusses the related approaches in task transfer techniques, centralized control systems overview and the state-of-the-art related to the real-time virtual machine. Section 3 presents the” Structured-Bytecode SBC and the generator engine. Section 4 introduces the proposed “Structured Bytecode real-time virtual machine SBC-RVM” design and implementation with a framework for task placement in a loosely-coupled computer system. Section 5 discusses the proposed algorithm and its implementation on real hardware with satisfactory results. Section 6 is the concluding remarks.

## 2 Related work

The proposed approach based on task transferee between nodes on loosely coupled computers especially in centralized control systems using an execution environment, which is a virtual machine. In this section, a state of art for those topics is discussed.

### 2.1 Task transfer techniques

Task transfer techniques were introduced to produce more processing power and resources’ sharing among processors on the network. The two types of task transfer are task placement and task migration. Task placement is defined as the transfer of a task which did not start yet, whereas task migration is the preemptive transfer of a task that had been started out but in a waiting state. The upshot of task transfer can be concluded, but not limited to Dynamic Load Balancing by migrating tasks from the overloaded node to a relaxed one [2]. Availability, which is moving off a task from the failed node to a healthy one. System Administration, which is the ability to migrate a task from the source node to another one for maintenance purpose. Fault Recovery, which is the procedure of stopping a task on the isolated faulty node, migrating to a healthy one and resume execution [3, 4]. When it is required to migrate a task from one node to another one, then both nodes should have a shared memory (i.e., shared address space) or common execution language. For Homogenous computer system, Common execution language such as machine code and assembly language can be sent to another node for remote execution. However, this technique is limited to that architecture and is not convenient in a loosely-coupled computer system where different computer systems are connected to a data bus as a network. In this case, an interpreted scripting language; like java byte-code or system emulator can execute the machine code [5] Many researches introduced various task transfer techniques for a different systems architecture, that are categorized as: Shared Memory Multiprocessors, where main memory is shared among all processors and Distributed Multi-Processors, where processors are on separate nodes [6]. Although task transfer is carried out between processors over a network, most of the implemented techniques were introduced for computer systems with a shared memory only, such as Grid computing [2], Cloud Computing [7], Heterogeneous/homogeneous multiprocessor system-on-chip (MP-SoC) [6, 8]. Unfortunately, there are no implemented techniques were introduced to support task transfer in the loosely coupled computer architecture.

The decision of migrating or placement of a task to a new host has two costs which are delay cost and migration cost. This optimization problem is proved to be NP-hard which can be converted to a weighted bipartite matching problem [9]. In a real-time application, the delay is acceptable if all tasks will meet the desired deadline.

### 2.2 Centralized control system

**Fig. 1:**
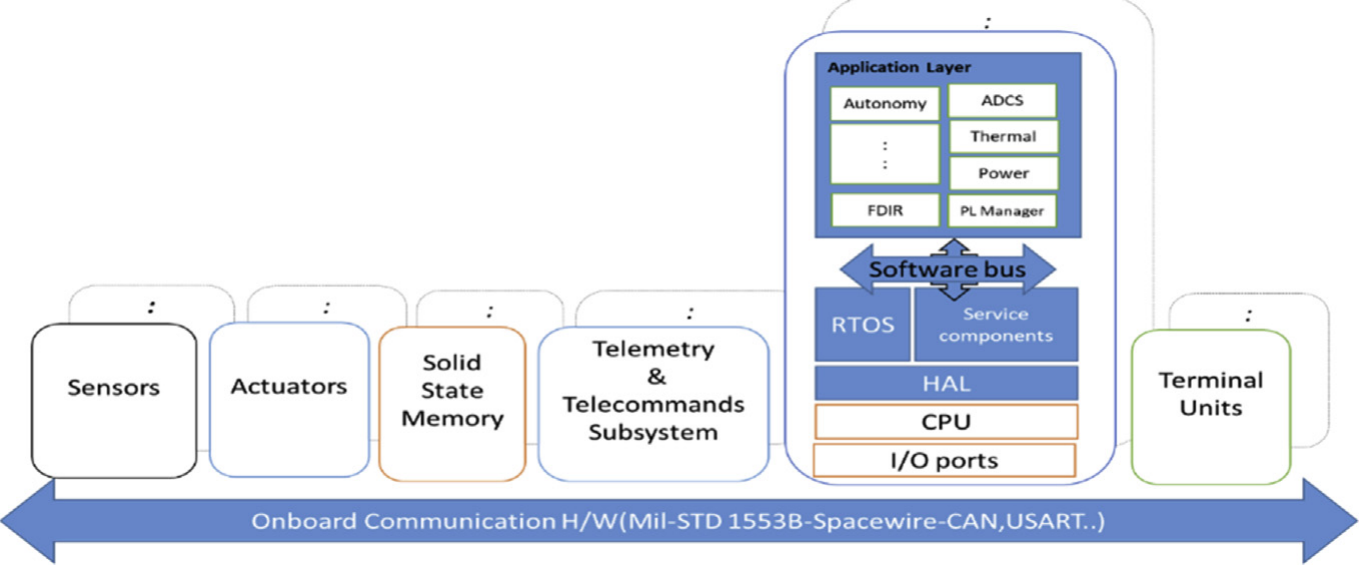
Typical OBC network -ESA OBCDH architecture.

Centralized control systems such as satellite control system, avionics, cruise missiles, and similar systems usually have a loosely coupled computer architecture [10]. The Central control unit controls all application tasks, manages data transfer over the network. These capabilities require high demand requirements for the onboard computer (OBC) and OBC-software (OBCSW) complexity. Spacecraft may travel in the deep space in a critical mission for years. Satellite control computer system; as shown in Fig. 1, consists of loosely-coupled computers connected via a common data bus such as SpaceWire, Military standard 1553, ARINC422, CAN buses.

Each computer came from various vendors with different architecture, processors, memory, and RTOS. It is required to have a common language to communicate with each other rather than exchanging data only. As long as the mission in space, a more off-nominal situation occurs and new features are required to be added. It is necessary to perform the desired concurrent control to the OBC and subsystems by accepting new remote tasks to be executed. Furthermore, if a piece of code could be sent to a subsystem over the network many of remarkable features will be added. The most interesting are: overcoming an off-nominal situation, solving off-design contingency remotely and adding new features. Therefore, overall system reliability is enhanced. This is the main motivation to introduce a new task placement technique in such systems where it is difficult to perform ordinary system maintenance or significant upgrading remotely.

### 2.3 Process real-time virtual machine

**Fig. 2:**
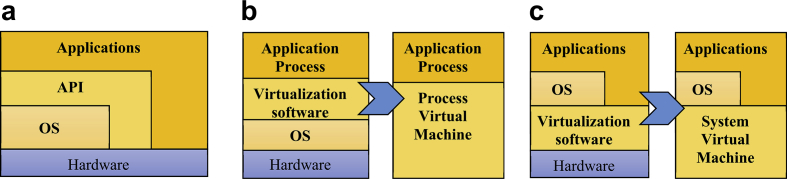
Different Virtual machines models.

In the beginning, the software was written for a specific instruction set architecture (ISA) and a specific operating system (OS). Applications layer communicate via the application binary interface (ABI) and application programming interface (API), where applications are bounded by the OS-ISA pair as shown in Fig. 2 a.

Process virtual machine (PVM) manages the run-time environment and overcome the OS-ISA pair limitation; as shown in Fig. 2 b by providing a higher abstraction level to execute code from different programming languages [11] on a different host machine. PVM provides a platform-independent environment for programming languages that interprets the code for an implicitly such as JVM [12]. The last model is the system virtual machine; as shown in Fig. 2 c, which is a lower virtualization level that the system platform or hardware is represented at a specific abstraction level. System-VM may host an operating system and applications together.

Most of the compilers that target embedded systems are platform specific. Limitations that are imposed when porting applications to a new platform appears. Thus, when a code is written for a specific machine, it becomes more challenging to be ported to another processor architecture and/or OS [13]. Some approaches tried to solve this problem such as cross-compilers capability to create a code which can run on another platform. The idea of the cross compilers is to reconfigure source code, which was developed for a specific platform into suitable code for the new host [14]. Compiled programs are bounded by the Application Binary Interface (ABI) to be operated for a specific OS and instruction set architecture pair, whereas PVM overcomes this limitation [15].

Virtualization in embedded systems shall satisfy real time requirements like timing constraints, performance and cost. Real-time virtual machines RVM is a research field that has many challenges such as worst-case execution time (WCET) analysis, porting on multiprocessor environment, time-predictable dynamic compilation [12, 16]. Another important challenge is VM in networked systems. Monolithic virtual machines are suitable for closely-coupled systems only, and far away to be applied to the modern networked system.

### 2.4 Java virtual machine

**Fig. 3:**
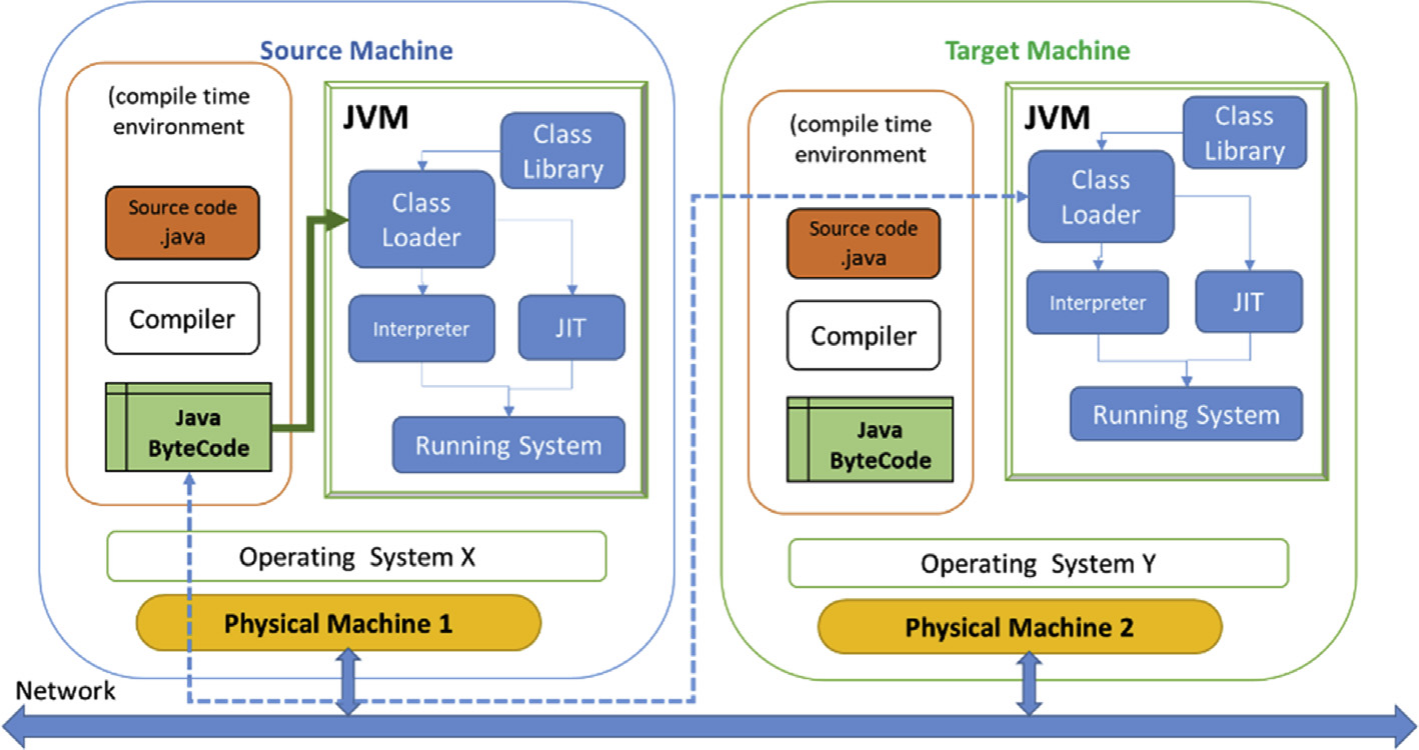
Java virtual machine task exchange on a loosely-coupled network.

Virtual machines differ in virtualization methodology and what to virtualize. Java Virtual Machine (JVM) abstracts the hardware and the machine to the developer [17]. This allows developers, not to concern on platform architecture. The code was written in Java should safely run on various platforms with JVM. The process is starting by translation of Java code into Java-bytecode as an intermediate machine-independent language as shown in Fig. 3. Java bytecode can be transfer over the network. On the target, JVM shall translate the bytecode into local machine native code to be executed. Hence Java's slogan, “Write once, run anywhere”. The just-in-time (JIT) compiles Java bytecode into a platform-specific executable code that is executed [18].

The overload of translating bytecode to the target machine native code limits the real-time capability for tasks immediately placement over the network. For that, and for java source language translation limitation, we were motivated to present a non-monolithic virtual machine for real time systems which run a unified code on any machine without retranslating and concerning about satisfying migrated tasks real-time requirements. This RTVM shall be used in long life centralized control systems such as satellite, nuclear plants, and similar systems, where subsystems are heterogeneous and running various RTOS. The proposed RTVM shall accept tasks written from different languages like C, Java, Python, etc., convert source code to a unified code which is able to run on a different machine without a need for re-compiling while preserving the required real-time constraints.

## 3 Design

### 3.1 Structured byte-code generator

Structured-ByteCode (SBC) generator converts specific source code or script with associated language grammar to another grammar called structured-bytecode. Similar to compilers, SBC generator performs the following functions: ‘Lexical Analysis,' which converts source code into tokens sequence, Syntax analysis to recover the syntax structure from the tokenizer and finally generates the “Intermediate Representations” (IR). SBC-generator converts source code into SBC in two steps, which are source code parsing and SBC generation.

#### 3.1.1 Source-code parser

**Fig. 4:**
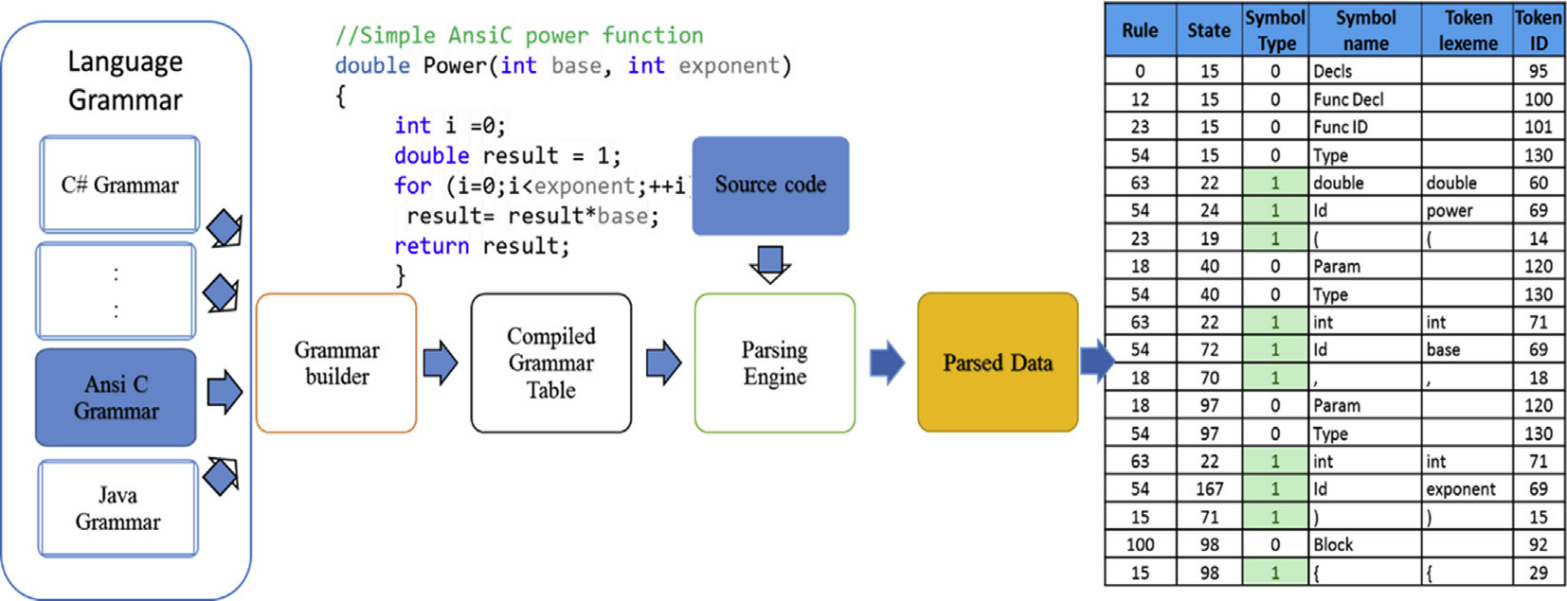
Parsing ANSI C source code by a compiled grammar file for “Power function.”.

Any programming language is composed of a set of grammar rules called productions, that are the syntax associated with this language. Production comprises terminal symbols and non-terminal symbols. Terminals are symbols in the source code such as reserved words, symbols, and identifiers. Each terminal is used to build up the Deterministic Finite Automata (DFA) to be used by the tokenizer. Nonterminal is the description of the terminal category in the grammar of the language such as statement, expression [19, 20]. Parser cut off the source code into grammatical records by a predefined grammar for that source code language and thus generate a special representation, which will be used in our generator. The parsing process is performed in three steps builder, Compiled grammar table, and the parser. Corresponding to the builder, target grammar is analyzed and creates a compiled grammar table file for the source code language. Parser engine reads both source files, compiled grammar tables and produces the parsed data as shown in Fig. 4.

#### 3.1.2 Structured byte-code generation

**Fig. 5:**
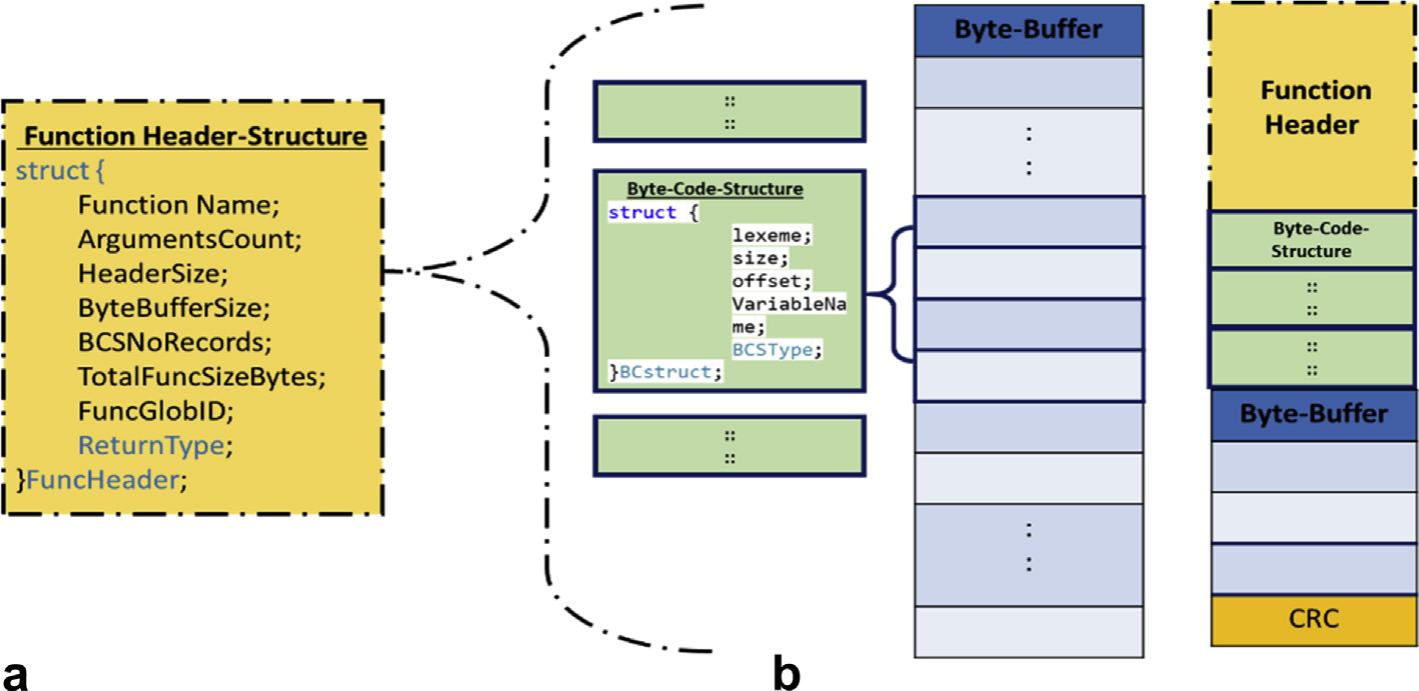
Structured Byte-code for a function.

SBC is constructed of three main components, which are Function Descriptor, Byte-Code-Structure, and Byte-Buffer as shown in Fig. 5 a. Function Descriptor (FD) is the representation of the smallest part of the code that can be placed/migrated to another computer on the network. FD presents the following information to a virtual machine on the host computer, which are a function name, number of arguments, Header size, count of records, size of Byte-buffer. The function name shall be unique across all computers on the entire network, and hence it has a unique ID by a combination of the system ID, source processor ID, and the function name as well. The second component is Byte-Code-Structure (BCS). BCS is a set of records representing one or more of the source code lines into SBC's representation. SBC-record represents one or more lexeme from the source code. BCS records are the instruction set of the proposed virtual machine (VM). The last component is Byte-Buffer (BB), which is the heap of the VM. At generation time, BB contains only the initialized variables. The format of the function is represented as a stream of bytes as shown in Fig. 5 b, where each byte is addressed by one or more BCS records.

##### 3.1.2.1 Scope

The scope of a variable, method or function is the parts of the code in which it is accessible. Scope concept varies from programming language to another but commonly has the concept of local and global scopes. A local scope is defined as accessible variables or functions at the current code block. Furthermore, the scope is defined as global variables or functions. Most programming languages support static scope only that specified by the code text, not at the runtime. Detection of incorrect variable dereferencing or function calling is the compiler rule. Nevertheless, at the code generation phase, SBC seeks only for the start and end of the scope. Furthermore, the next block of statements will be accessible or not. Scanning the code is done by a decision-making a tree. The token may be a function, variable declaration, statement, etc. A function, for example, will be processed as follows: find the scope (start and end), return type and function arguments. If the function has an argument, the next token may be another argument or another token type. The recursive search of the entire scope gives the possibility to go from the tree root-leaves-root for every token and results in a full scope transformation into SBC.

##### 3.1.2.2 SBC presentation

**Fig. 6:**
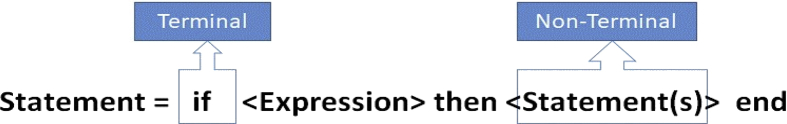
Parsing code into terminals and non-terminals.

The parsed source code is formed of tokens, which can be a terminal or statements. The statement is a set of terminals and non-terminals tokens as shown in Fig. 6. Non-terminals are syntactic structures that are defined by the used language grammar.

Terminals are any defined object like reserved words, defined variables, operators, sign, numeric, string, etc. Each terminal is represented as SBC record. Each record has four fields which are lexeme, size, offset and name. A lexeme is a terminal name; Size is the number of bytes used by that terminal in the BB, offset from the start of the buffer; Variable-name is the ID of that variable (in the case of a variable). The statement may comprise a set of terminals. SBC-generator has three types of statements that are data representation, flow control statement, operation statement. Combinations of terminals in one statement are unlimited and may have an unlimited number of operators and operands. For that reason, the first instruction in a statement is constructed after later instructions were defined. The following sections are a demonstration of some SBC instructions for ANSI C language.

##### 3.1.2.3 Data representation

**Fig. 7:**
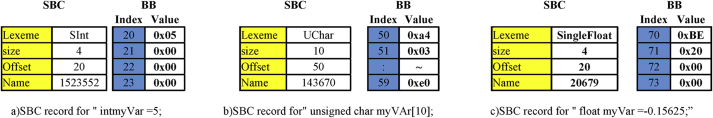
SBC Data representation examples.

Data is represented by the compiler according to the target processor architecture and OS. Data representation may vary according to the target platform in byte order little or big endianness, memory alignment, floating point representation, etc. In SBC generator, Name of data (variable, constant, etc.) is a unique ID by combining task and variable names as a numerical value. SBC VM according to the lexeme of the variable knows what each byte in BB should represent. As shown in Fig. 7 an example of ANSI C data types is represented as SBC records.

##### 3.1.2.4 Flow control statement

**Fig. 8:**
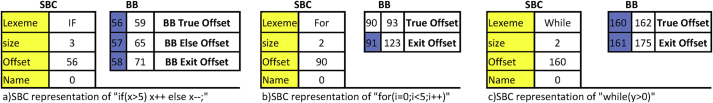
Example of flow control representation of ANSI C in SBC format.

Flow control instructions vary from one programming language to another in presentation and structure, whereas the concept remains the same. Conditional statements such as *If* and *Switch* statements, Loop statements like” *for” and “while” loops* are represented in a simple structure on SBC as shown in Fig. 8.

##### 3.1.2.5 Operation statement

The statement is the smallest brick of programming language structure, which expresses an action(s) to be carried out. Operations like add, subtract, increment, decrement, jump, etc. Operation statement comprises one or more statements. For example,” x*+=a+b*” can be divided into” a*+b*”,” *x+(a+b)”* and an assignment statement “*x=sum of all*.” These varieties may add complexity to the generation of SBC code when it is parsed from left to right. Decision tree makes it easy to accumulate all instruction on the root statement which is an assignment statement “x = ”. Thus the Operation statement is represented by a set of SBC's records. Operation statement can be a function call; this function will be transformed into a sequence of SBCs in the primary function for portability purpose.

### 3.2 The structured byte-code real-time virtual machine

#### 3.2.1 Definition and architecture

**Fig. 9:**
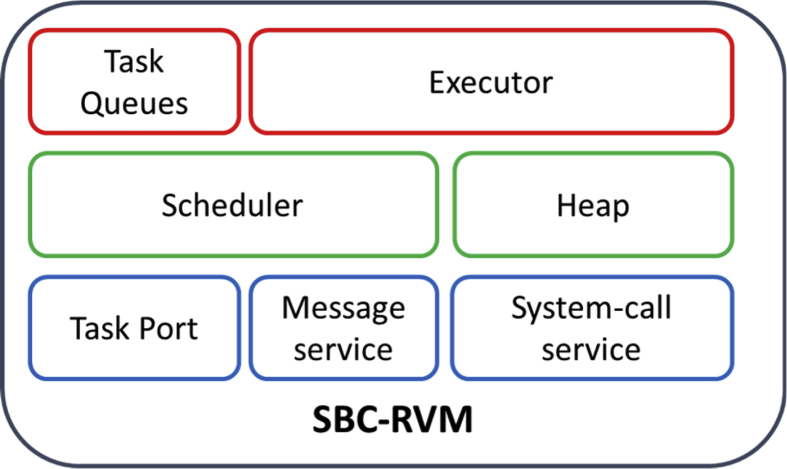
SBC-RVM architecture.

**Fig. 10:**
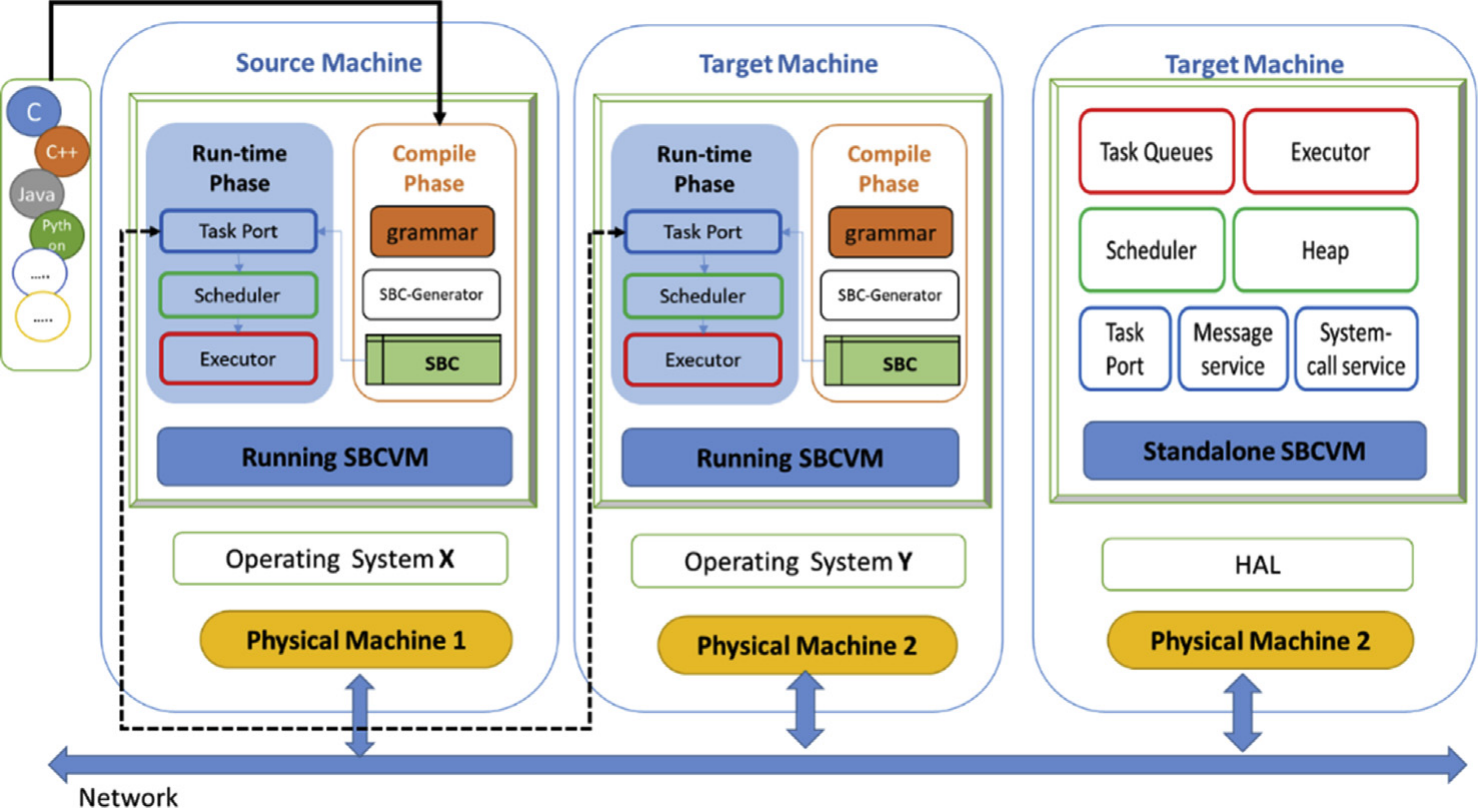
SBC-RVM on a loosely-coupled network.

The SBC Real-time Virtual Machine (SBC-RVM) is the execution platform of SBC tasks, which operate on the local machine or have been migrated from an original node to be placed on the host machine. SBC-RVM architecture, as shown in Fig. 9 is composed of three layers. The lower layer performs low-level functions such as task port, message service, and system-call service. Task port inspect and accept new tasks from the network. Message service exchange message between SBC-VMs on different nodes. System call service handles system call with the host OS. The intermediate layer is for scheduling of tasks from “Task Port” to be placed in the corresponding queues and the “heap management” for different tasks at the execution time. The upper layer is formed of Task-queues with different priorities and frequencies and the Executor of the tasks instants into the Heap. SBC-RVM is represented in two forms, which are standalone form, the second as an application at the application layer which is hosted by an RTOS as shown in Fig. 10. The efficiency of the OS-VM pair can be improved by adding the property of communication and cooperating; this property called Para-virtualization [4, 21].

SBC-RVM and JVM as shown in Figs. 3 and 10; differs in the following: SBC-RVM translates code from any language to SBC form automatically using provided language grammar, whereas java translates only Java code. The second one, SBC does not need to the in-time translation of SBC to the running machine native code. SBC-RVM executes tasks on SBC form. This difference gives SBC more credit on the run-time environment and portability issues.

#### 3.2.2 Runtime mechanism

The basic function of SBC-RVM is scheduling, executing local or migrated tasks and exchange messages such as results and acknowledgment with other SBC-RVMs on the network. Furthermore, provide handshaking with other real time VMs over the system bus. The migrated task τ_n_ is accepted at the host machine by “Task port” service, which inspects incoming task for data integrity and hospitality. The task is accepted when its real-time constraints match scheduler requirements and target processor utilization. The used scheduler is a multilevel-queue with different frequencies and priorities. Scheduler picks up the right task to the “Executor” from the associated queue to be loaded into the heap for execution. Executer loads SBC instructions sequentially into the heap. The return, if any, sent back to the origin node via the “Message service.”

#### 3.2.3 System call service

The guest OS and the RT-VM can communicate to support the VM with related RTOS activities. SBC-RVM operates at the hosted RTOS with user-level permissions (unprivileged) and all SBC-RVM system calls are mapped to the host RTOS system ones.” System calls services” include interrupt-handler, I/O peripherals read/write, timers set/reset, etc. It is the only platform dependent part on SBC-RVM. By maintaining an integrated set of interfaces, SBC-RVM can interact with the OS and can be easily modified to support alternatives platforms [13]. In standalone form, hardware abstraction layer (HAL) should be modified to support different target platforms.

#### 3.2.4 Message service

Exchange messages between different nodes that are running SBC-VM in a loosely-coupled environment are mandatory. Thus three different types of messages are implemented that are Task exchange messages, Information messages, and Service messages. The message header contains the preserved real-time constraints from its origin node. Each message type can be in “Broadcast” or “Direct” message formats. A broadcast message where source node can send a message to all nodes on the network, whereas direct message is between the source and the destination node. “Task exchange” type is used for SBC-task transfer between nodes on the network. “Information messages” such as a result of the migrated task to origin node, acceptance, acknowledgment, etc. “Service Messages” are these messages that contain commands from one VM to another like delete, pause a periodic task execution.

#### 3.2.5 Task port

SBC-RVM conform to the environment to support SBC task placement while preserving real-time constraints. The migrated task is accepted by the host VM with a grantee to fulfill its real-time properties. Tasks' requirements are attached to the “Task exchange” message header. The requirements are worst-case execution time (WCET), execution rate and deadline properties. To guarantee the required temporal behavior at the host node, a static mechanism is implemented for enforcing the required behavior whenever it is possible. This approach can be accomplished by knowing the WCET a priori noting that it strongly depends on the used programming language, origin processor architecture and the platform-compiler optimizations [22]. The SBC-RVM scheduler computes the execution time of the running tasks continuously while saving the last execution time and the WCET i.e. the maximum execution time ever. At the host node port, migrated task's real-time requirements is inspected by the “Task port”, where tasks with a predictable behavior that can be fit at the host are only accepted [23, 24]. The migrated task τ_n_ (*C*_n_, *D*_n_, *R*_n_) is then characterized by its run-time properties, where *C*_n_, is execution time, *D*_n_ deadline and *R*_n_ is the arrival rate of the n^th^ task. The inspected task is accepted and then assigned to the appropriate queue Q_x_ in “Task Queues” if Q_x_ can preserve its runtime properties.

#### 3.2.6 Scheduler

SBC-RVM scheduler is a multilevel queue scheduler which was presented in [25] and named “SMAMLQS”. SMAMLQS has four queues of different priorities and frequencies. The queues' internal scheduler is Early Deadline First (EDF) scheduler. Each queue is for a specific type of tasks, which are hard real-time queues *Exchange tasks* “ET” queue and *Periodic tasks* “PT” queue, *Soft Real-time tasks* “ST” queue and *Background tasks* “BT” queue. The scheduler executes each queue according to predefined frequencies and priorities. SBC-RVM has a period and deadline (*P*_SBC_, *D*_SBC_) which are assigned by the host RTOS or is configured in the standalone form. SBC-RVM's scheduler calculates the utilization of real-time queues *U*_ET_, *U*_PT_, *U*_ST_ according to Eq. (1) where *C*_i_ is the execution time of *Q*_i_ over the period *P*_i_. Total utilization *U*_SBC_ is calculated according to Eq. (2).

(1) $$U_{\text{i}}\;=\frac{C_{\text{i}}}{P_{\text{i}}}\;\;\leq U_{iCr}$$

(2) $$U_{\text{SBC}.\text{m}}\;=\frac{C_{\text{ET}}+C_{\text{PT}}+C_{\text{ST}}}{P_{\text{SBC}}}\;\;\leq U_{Cr.m}\;\;$$

SBC-RVM is tending to maintain a safe utilization level called *Critical Utilization-Level U*_Cr,n_ at node n. The scheduler has the sufficient condition for successful scheduling whenever *U*_SBC,n_ ≤ *U*_Cr,n_ and *0* < *U*_Cr_ ≤ 1. When *U*_SBC_ exceeds the critical level *U*_Cr,m_, the scheduler requests to place a selected task(s) to one of the neighbor nodes on the network to maintain the load balancing for example. This can be done by configuring SBC-RVM to pick task(s) from one of lower priority queues. For administration purpose, a command to place a task from a node to another can be issued. For a task τ_n_ at origin node n, the Selection of the destination node can be done in two ways: “Appeal” broadcasted message to all nodes on the network includes tasks header as τ_n_ (*C*_n_, *D*_n_, *R*_n_). The first responder is the node which can fit τ_n_ real-time requirements. Origin node then sends SBC representation of τ_n_. The second way is a “Direct” message to a destination node on the network chosen from a look-up table, where preferred destination nodes are sorted. The acceptance criteria at “task port” of a migrated task **τ**_n_,_m_ (sporadic, batch, or periodic) are given by Eq. (3), where *P*_*i*_ is the period of task i.

(3) $$U_{Q,m}+\frac{C_{n}}{P_{i}}\leq \;U_{QiCr.m}\;\forall \tau_{n}\in Q_{i.n},\;Q_{i}\in \left\{Q_{\text{ET}},Q_{\text{PT}}\;,Q_{\text{ST}}\right\}m$$

#### 3.2.7 Heap management

Protection of operating memory is one of the leading issues in safety-critical systems which operates RTOS and RT-VM [26]. SBC-RVM is characterized by a predefined and concrete memory manipulation mechanism. This mechanism may add overload on SBC generation but ensure the predicted results without any dynamic memory allocation and deallocations. The chosen task's Byte-Buffer BB is uploaded to the heap at execution time, which contains the initialized variables and constants only, whereas local variables are allocated on the heap during execution. This approach has a disadvantage in the amount of memory used by each task. At the end of task execution, it is uploaded from the heap. This effect is minimized due to the sequential execution of SBC tasks instant.

#### 3.2.8 Task Queues

SBC-RVM has four main queues, which are *Exchange tasks* “ET” queue and *Periodic tasks* “PT” queue, *Soft Real-time tasks* “ST” queue and *Background tasks* “BT” queue. Each queue has sub-queues with different frequencies. Local tasks are assigned to each queue according to its type. New migrated tasks are classified and assigned to one of the four queues. Tasks’ priority is sorted in each queue by the Earliest Deadline First (EDF) scheduling policy. After flushing each queue, its utilization is then calculated and will be considered as acceptance criteria for the new sporadic or periodic task at “task port.”

#### 3.2.9 Executor

Executing a task starts by analyzing function header, load non-initialized variables to the heap. The first SBC record represents a flow control or operation statement as described in 3.2.2. The function argument (values if any) are loaded to the corresponding SBC record. The Executor has the same content as instruction pointer IP register where the index of the currently executed SBC record is stored. Execution continues till the last SBC record or “STOP” command is hit which is similar to “return” command. A Task successful-execution message with the return value is then passed to the “Task Port” module to be sent to the origin node.

## 4 Results and discussion

To validate the proposed approach, two experiments were performed. The first one is to prove the concept of SBC versus original code in terms of performance and results' correctness [27]. The second one is to realize the concept of task placement support on the loosely-coupled network using SBC-RVM to measure its applicable potential on such complex systems.

### 4.1 SBC performance evaluation

**Fig. 11:**
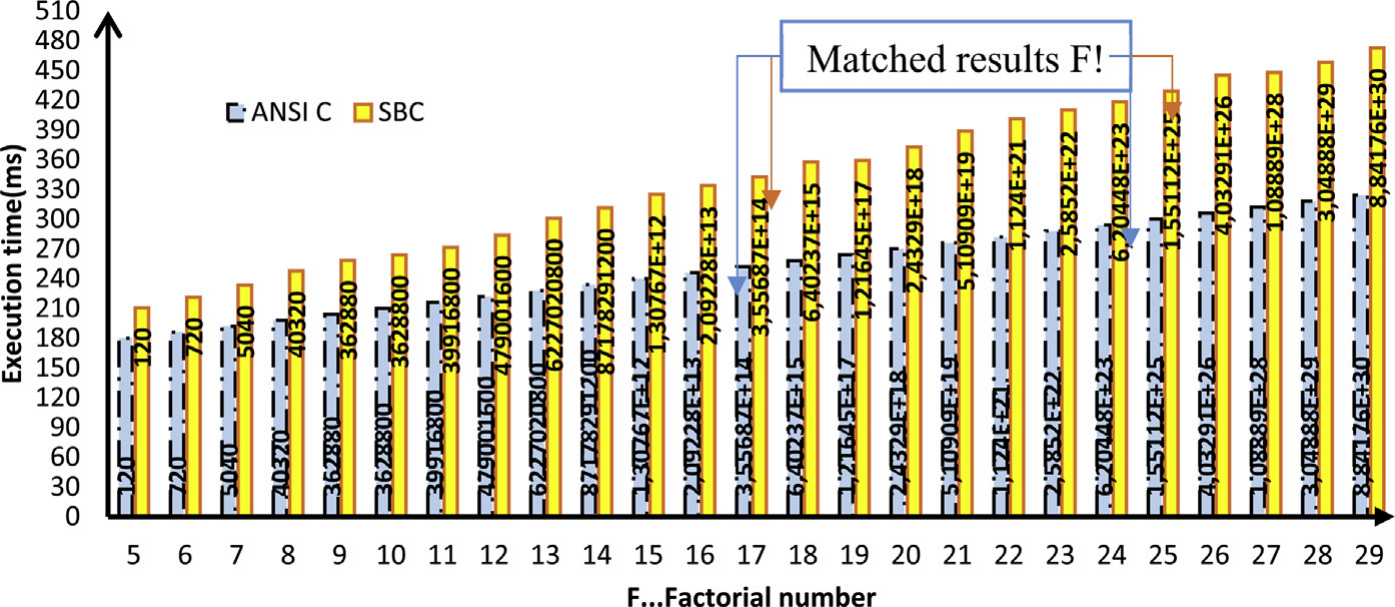
Performance Evaluation for SBC versus ANSI C factorial.

**Fig. 12:**
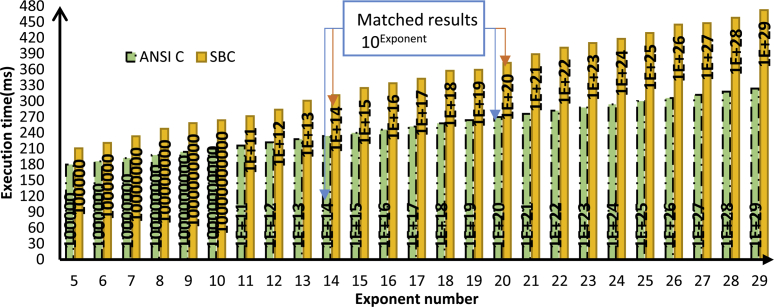
Performance Evaluation for SBC versus ANSI C Power function.

To evaluate the SBC-RVM performance, it will be compared in execution against native code [27]. The performance was measured by benchmarking using two functions were implemented in ANSI C as a source-code language to prove the concept of SBC language. Thus, the SBC performance benchmark is evaluated at run-time. The functions are to compute the factorial and the power of any given number. “SBC generator” transforms the two functions into SBC format. SBC generator runs on the μVision IDE – Keil for ARM cortex M4 target platform. The test runs on the evaluation board STM32F407VGTx, Core ARM Cortex-M4, FPU MPU 168 MHz, Memory 192 kB RAM, 1 MB ROM, Clock & Power 1.80 V,3.60 V. The experiment starts by running each function (SBC/ANSI C) with a rising base number. The results as shown in Figs. 11 and 12. Each function is executed under different workloads by the tested SBC-RVM to evaluate the relative performance between native code (ANSI C) and SBC-representation for the same code under the same environment. During the test, the argument of each function is incremented to represent the performance of the two approaches.

The performance evaluation of Java versus C++ shows that java is slower [28]. states that Java is 2 times slower using a modeling benchmark. The experiment shows that SBC performance is sufficient comparison to the native code. The execution time as shown in Eq. (4).

(4) $$C_{\text{i}\;\left(\text{SBC}\right)\;}\;≅\;1.4\text{*}C_{\text{i}\;\left(\text{ANSI}\;\text{C}\right)\;}\;$$

### 4.2 SBC server-client test

**Fig. 13:**
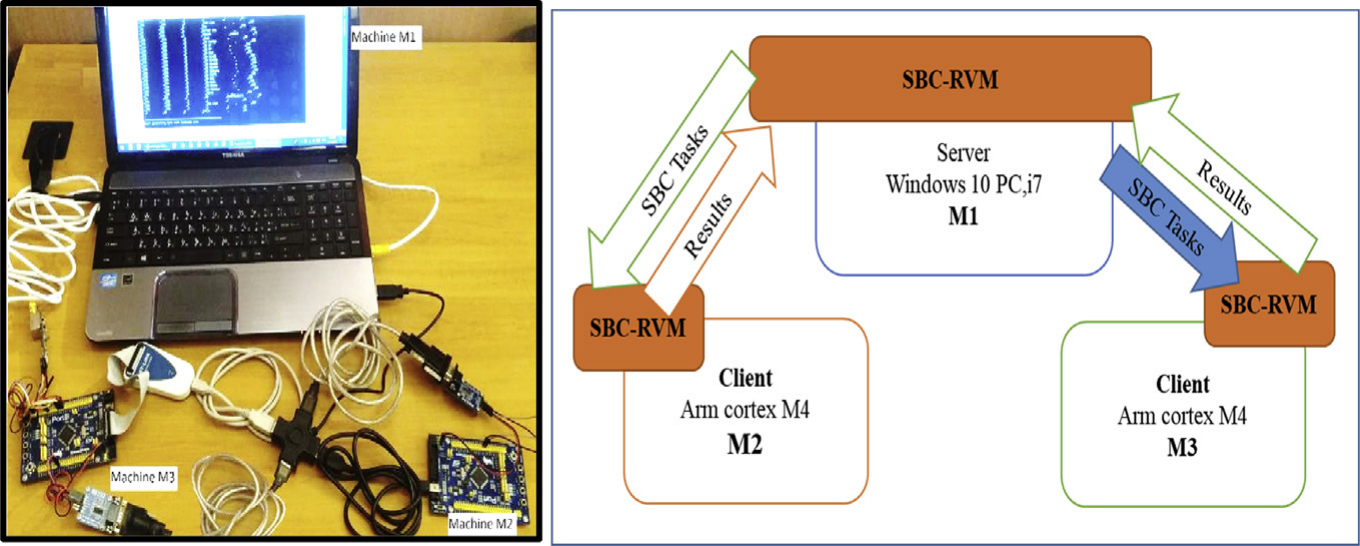
SBC server-clients in a star network experiment.

The second experiment had been run for the same platform conditions. The setup of three machines connected in a star topology using serial data bus RS232 to simulate the loosely-coupled environment. The first machine running Windows 10, Intel i7/2 cores/2.4 GHz each, with 8 GB of RAM; SBC-RVM should be run with the highest priority level. The second and third machines are ARM Cortex-M4 core with FPU, 1 Mbyte Flash, 168 MHz CPU. The second Machine operates 168 MHz with the RTOS's scheduler presented in [25], which the used scheduler is a multilevel-Queue scheduler configured with four different queue priorities. SBC-RVM is represented as a hard real time task that operates with a period of 200 ms and it has the highest priority with a 400 ms deadline. The third machine operates at 100 MHz and runs SBC-RVM with a hardware abstraction layer on a typical machine with the second one. The three different machines are connected by a data bus as a loosely coupled computer system. This experiment tests for Server-client framework to support task placement in loosely-coupled computer systems using the proposed SBC-RVM as shown in Fig. 13. Consider a given set of tasks T:{τ_i_: i ∈ [1: *N*]} with different priorities and frequencies and distributed over the four queues. A task τ_m,n,q_ at machine M1 should be replaced on another machine m as τ_m_,_n,q,t_ on the network and the execution results should be sent back to the server to meet τ_n,q_ deadline. In order to minimize the peak resource usage while preserving real time constraints, every migrated tasks deadline must be achieved. Communication cost, WCE of the migrated tasks is known in advance on the origin machine. The decision of task replacement to another machine on the network should be known in advance. Otherwise, tasks real time constrains could not be met. All the tasks can meet deadlines and the peak resource usage is minimum among all the feasible solutions [29].

The communication cost is neglected for simplification where:

• m… machine number, m = {1,2,3}, n…task number, n = {1,2, 3,..,10},q… Queue of task τ_n_ = {Q1: highest priority Queue ET (f:10 ms, d = 100 ms), Q2-AT (f:100 ms, d = 200 ms), Q3 = ST (f:1s, d = 2 s), Q4 = BT (sporadic, d = non) }. o t…the trial number of the task. o Task pool is five tasks of each queue level.

In this experiment, only one machine M1 generates SBC for any task τ_m_,_n,q,t_ and request to place τ_n,q_ based on specific migration criteria over the network. At the start, Machine #1 generates an SBC for τ_n,q_ (written in ANSI C) and request to place τ_m_,_n,q,t_ as follows:

• M1 continuously generate task τ_m,n,q,t,_ and request to place on M2 periodically according to each task arrival rate.
• In case of adverse reply from M2, M1 starts to route tasks to M3 for the rest of tasks belongs to the same queue level.
• The experiment stops when a negative reply message from M2, M3 for each queue levels 2,3.

**Fig. 14:**
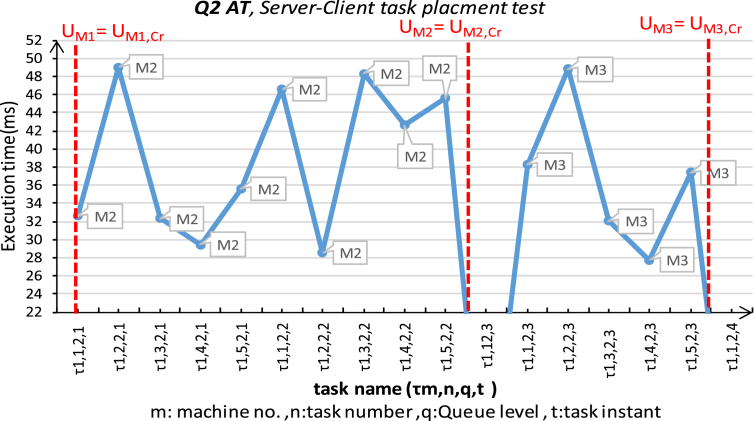
Server-Client (M1: M2, M2) task placement for “Application task AT-Q2” for load-balance on M1.

**Fig. 15:**
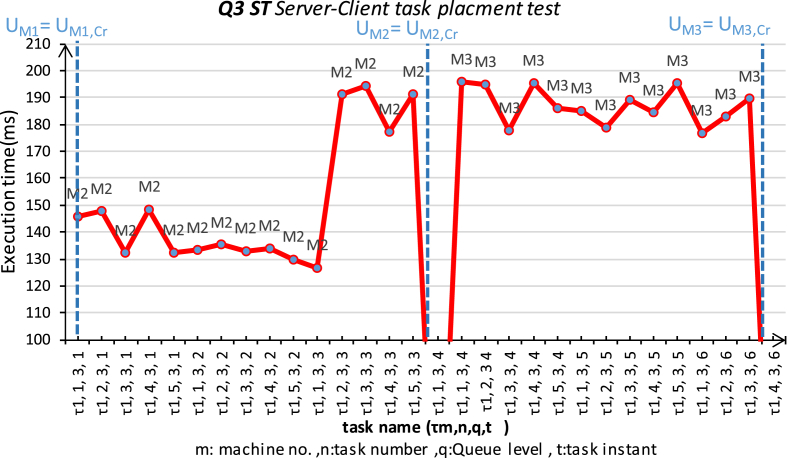
Server-Client (M1: M2, M3) task placement for “Soft Real-time tasks ST-Q2” for load-balance on M1.

The request and replied result times for each task is monitored and recorded. The results are shown in Fig. 14 for Q2 tasks and Fig. 15 for Q3 tasks. The two figures show satisfactory results for placing those real time tasks over a loosely-coupled network using the proposed SBC-RVM while preserving real-time constraints of the placed tasks. The server M1 simulate an overloaded node and start requesting to place tasks from Q2, Q3 on M2. The experiment continues to tell U_M2_ = U_M2, Cr_. At this moment, SBC-RVM cannot preserve real-time properties of any new tasks and M1 starts to send appropriate tasks to M3 until U_M3_ = U_M3, Cr_.

**Fig. 16:**
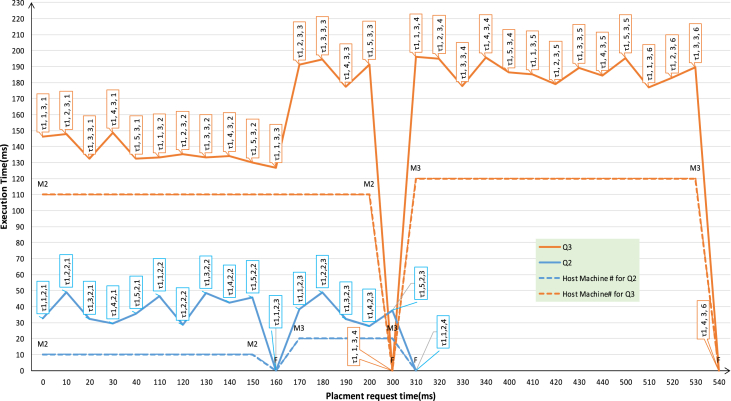
Server-client task placement rate for Q1,2 on M1,2.

The experiment was held using a Server-client framework where all tasks met their deadline with the right logic. Fig. 16 shows the arrival rate of tasks from both Q2, Q3 from M1 to M2 and M3 respectively. It is clear that as the number of nodes on the network operates SBC-RVM, the more reliability, load sharing, and new features can be added to that system.

## 5 Conclusion

Structured-byte code real-time virtual machine (SBC-RVM) is proposed to support task placement in loosely-coupled computer systems such as satellites, military systems, and similar control systems. Those systems are characterized by long life, hard environment, and remote control operation. SBC-RVM is introduced to add more features, control, and administrations without a need for software upgrading. SBC-RVM runs a platform independent code called SBC, which is generated automatically from source code using its native language grammar. Unlike Java virtual Machine, SBC accepts tasks which were written in any language with a known grammar to be executed on any platform-OS pairs without a need for interpreting again to the new machine code. The proposed SBC-RVM can exchange tasks and messages over the network to support task placement for different goals such as load balancing, sharing, fault recovery, administration, software-voting, and remote commands execution. SBC-RVM includes a multilevel queue scheduler for classifying local and new placed tasks according to priorities and frequencies to the appropriate queue, whereas the inner queues’ scheduler is Earliest Deadline First (EDF). The concept and performance of SBC are proven and evaluated versus the original code and shows a satisfactory result. SBC-RVM simplifies the communication between nodes, meet tasks real-time constraints, help to relax overloaded nodes, adding new tasks to the service, issuing remote commands to remote systems without a need for a significant upgrade. The proposed techniques showed promising results to support task placement over loosely-coupled real-time computer systems while preserving the real-time properties of the placed tasks. The SBC-RVM shows a possible potential for a real-time virtual environment and can be applied successfully to that kind of real-time systems. In case of future research, it should be appropriate to test more languages and scripts for more evaluation of SBC-RVM and its adaptability to different platforms.

## Declarations

### Author contribution statement

Mohamed O. Elsedfy: Conceived and designed the experiments; Performed the experiments; Analyzed and interpreted the data; Contributed reagents, materials, analysis tools or data; Wrote the paper.

Wael A. Murtada: Conceived and designed the experiments; Performed the experiments; Contributed reagents, materials, analysis tools or data; Wrote the paper.

Ezz F. Abdulqawi: Analyzed and interpreted the data; Contributed reagents, materials, analysis tools or data; Wrote the paper.

Mahmoud Gad- Allah: Contributed reagents, materials, analysis tools or data; Wrote the paper.

### Funding statement

This research did not receive any specific grant from funding agencies in the public, commercial, or not-for-profit sectors.

### Competing interest statement

The authors declare no conflict of interest.

### Additional information

No additional information is available for this paper.
